# Bioleaching of Arsenic-Rich Gold Concentrates by Bacterial Flora before and after Mutation

**DOI:** 10.1155/2013/969135

**Published:** 2013-12-05

**Authors:** Xuehui Xie, Xuewu Yuan, Na Liu, Xiaoguang Chen, Awad Abdelgadir, Jianshe Liu

**Affiliations:** College of Environmental Science and Engineering, Donghua University, 2999 North Renmin Road, Shanghai 201620, China

## Abstract

In order to improve the bioleaching efficiency of arsenic-rich gold concentrates, a mixed bacterial flora had been developed, and the mutation breeding method was adopted to conduct the research. The original mixed bacterial flora had been enrichedin acid mine drainage of Dexing copper mine, Jiangxi Province, China. It was induced by UV (ultraviolet), ultrasonic, and microwave, and their combination mutation. The most efficient bacterial flora after mutation was collected for further bioleaching of arsenic-rich gold concentrates. Results indicated that the bacterial flora after mutation by UV 60 s combined with ultrasonic 10 min had the best oxidation rate of ferrous, the biggest density of cells, and the most activity of total protein. During bioleaching of arsenic-rich gold concentrates, the density of the mutant bacterial cells reached to 1.13 × 10^8^ cells/mL at 15 days, more than 10 times compared with that of the original culture. The extraction of iron reached to 95.7% after 15 days, increased by 9.9% compared with that of the original culture. The extraction of arsenic reached to 92.6% after 12 days, which was increased by 46.1%. These results suggested that optimum combined mutation could improve leaching ability of the bacterial flora more significantly.

## 1. Introduction

With the development of economy, high-grade gold ores are almost processed, low-grade gold ores and concentrates are becoming the main gold resources. Thus, it is increasingly necessary to process ores of lower grade to meet demand, especially on arsenic-rich gold concentrates [1, 2]. In arsenic-rich gold concentrates, gold grain is always occluded by sulfide minerals such as arsenopyrite (FeAsS), pyrite (FeS_2_), realgar (As_2_S_2_), or orpiment (As_2_S_3_), which severely cut off the cyanide from gold grain [3, 4]. To achieve a satisfactory extraction of gold grain, an oxidative treatment is required to break down the blockade of sulfide minerals before cyanide leaching [5]. Bacteria oxidation has the advantages of low cost, low energy consumption and environmental protection, which shows the broad prospects for development on the treatment of low-grade gold ores and concentrates [6, 7]. However, bioleaching bacteria have some drawbacks such as long growth cycle and slow oxidation activity, which lead to poor effect on bioleaching [8].

Due to the major drawbacks of bioleaching bacteria, selecting and breeding the efficient bacteria for gold concentrates bioleaching are becoming more and more important. Mutation breeding is a frequently-used and effective method of obtaining excellent leaching microorganism. Xu et al. [9] used UV mutation to treat strains *Acidithiobacillus ferrooxidans* GF and *Acidiphilium cryptum* DX1-1 for chalcopyrite bioleaching, which obtained excellent leaching bacteria and increased the dissolution of copper. Kang et al. [10] treated mixed microorganisms with mutagens NO_2_^−^, diethyl sulfate (DES), UV, and their combinations, and the best one increased the content of Cu^2+^ by 101.4% in 20 days of leaching compared with the control culture on chalcocite-leaching.

Many studies on mutation breeding of bioleaching bacteria have been done all over the world, but few focused on the bacteria for arsenic-rich gold concentrates bioleaching [11, 12]. In this paper, the bacterial flora enriched in acid mine drainage of Dexing copper mine, Jiangxi Province, China, was used to be mutated and for arsenic-rich gold concentrates bioleaching. UV, ultrasonic, and microwave were major mutation methods and were adopted alone or combined to obtain excellent bioleaching bacteria. The effects of mutation on bacterial growth, activity and arsenic-rich gold concentrates bioleaching were the main contents in this study.

## 2. Material and Method

### 2.1. Bacterial Flora

Original bacterial flora was enriched in acid mine drainage of Dexing copper mine, Jiangxi Province, China. Microbial diversity of the bacterial flora had been analyzed by RFLP analysis [13, 14]. Results indicated that the bacterial flora was dominated by moderate thermophilic microbes, including *Leptospirillum *sp. and *Sulfobacillus* sp. two putative divisions. Especially, *Leptospirillum *sp. was the most dominant division, which represented 52.5% of the total clones in the 16S rDNA cloning library of the bacterial flora (data not shown).

### 2.2. Mineral Sample

In this study, the arsenic-rich gold concentrates were obtained from Shandong province, China. The element analysis results of the sample were shown in Table 1.

It could be seen that the sample was rich of arsenic (As 9.3%) and belonged to arsenic-rich gold concentrates. X-ray diffraction (XRD) patterns of arsenic-rich gold concentrates showed that the main mineral phases were arsenopyrite (FeAsS), pyrite (FeS_2_), quartz (SiO_2_), and gypsum (CaSO_4_ *·*2H_2_O). The mineral sample was grounded and the particle size was <74 *μ*m for bioleaching experiments.

### 2.3. Culture Medium

The bacterial flora was cultivated in 9 K liquid medium. 9 K liquid medium: (NH_4_)_2_SO_4_ 3.0 g/L; MgSO_4_ *·*7H_2_O 0.5 g/L; K_2_HPO_4_ 0.5 g/L; KCl 0.1 g/L; Ca(NO_3_)_2_ 0.01 g/L, and FeSO_4_ *·*7H_2_O was the energy source for bacterial growth. pH value of culture was modulated to 1.5 with 5 mol/L H_2_SO_4_. The liquid medium was sterilized at 121°C for 20 min before use. Bacteria were inoculated in 250 mL flasks containing 100 mL 9 K medium. Flasks were incubated at 40°C in a rotary shaker at 160 rpm.

### 2.4. Mutation

Cells in the logarithmic phase were centrifuged for 20 min (10000 rpm) and suspended in 9 K basic salts medium without Fe^2+^, and the density of cells was adjusted to about 1.8 × 10^8^ cells/mL.

Mutation experiments were performed under the following conditions. For UV mutation, the power of UV lamp was 15 W. The suspended cells were taken into a plate and at a distance of 30 cm to the UV lamp, and the radiation time was 60, 120, and 180 s, respectively. After mutation, the sample was kept away from light and stored in the refrigerator at 4°C for 12 hours to prevent the bacteria from recovery with light. For microwave mutation, the bacterial suspension was treated in a microwave oven (2450 MHz, 700 W) for 10, 20 and 30 s, respectively. Bacterial mutant were transferred on the ice to remove thermal radiation, and protect enzyme from inactivation. For ultrasonic mutation, ultrasonic cells crusher was used to treat the suspended cells. The power was 500 W and frequency was 40 KHz; the treatment time was 10, 20, and 30 min, respectively. For combination mutation, UV, ultrasonic, and microwave were used, respectively, to two combinations and three combinations for composite mutagenesis. After mutation, the bacterial mutants were cultivated in 9 K liquid medium under optimal growth conditions, and the density of cells and rate of Fe^2+^ oxidation were measured every 12 hours. The bacterial mutants with good oxidation activity on Fe^2+^ were selected in gold concentrates bioleaching experiments.

Total protein activity detection. The bacterial mutants selected growing at the stationary growth stage in 9 K medium were collected by centrifugation (10,000 rpm for 20 min), washed with distilled water, and suspended in the acetate buffer. pH value of suspension was adjusted to about 5.8. Cells obtained were crushed by biological cell disruption of AH-100B (ATS Engineering Inc. Canada) and centrifuged at 10,000 rpm for 30 min (4°C) to collect total protein. The total protein activity was determined by two-point inverse calibration method [15]. In this study, the amount of ferrous ions oxidation in unit time was used to weigh the activity of total protein, and a purple coordination compound was formed by combining ferrous ions nonoxidized with ferrozine. Total protein activity was monitored by measuring the absorbance of the compound in 570 nm wavelength using a UV-vis spectrophotometer (Hitachi Model U-2910). The less absorbance of the compound, the more activity of bacterial total protein.

### 2.5. Bioleaching Experiments

Bioleaching experiments were conducted in 250 mL flasks containing 100 mL 9 K liquid medium and 10% (v/v) of bacterial flora. The pulp density of arsenic-rich gold concentrates was 5% (w/v), and the initial pH of the culture was adjusted to 2.0 with 5 mol/L H_2_SO_4_. Flasks were incubated at 40°C and 160 rpm in a rotary shaker. During bioleaching process, the density of cells, pH values, concentration of total arsenic, and total iron in the solution were determined at certain intervals. The medium in flasks taken out must be compensated with 9 K medium, and the evaporation of water in flasks was supplemented with distilled water. All experiments were performed in triplicate and the average values were reported.

The density of cells was determined by hemacytometer counting method under the optical microscope (Olympus CX31). Potassium dichromate titration was used to calculate ferrous iron oxidation rate. Concentrations of total arsenic and total iron were determined by ultraviolet spectrophotometry. pH values of the solution were measured by multifunctional water quality analyzer (WTW, Germany).

## 3. Results and Discussion

### 3.1. Mutation Results

Considering that bioleaching bacteria was strictly chemoautotrophy bacterium, the existence of organic matter would inhibit its growth [16]. And furthermore, the majority of chemical mutagens play the best mutation effect only in the near-neutral environment, but decompose into a variety of harmful organic compounds in the acidic environment, so, physical mutagens such as UV, ultrasonic, and microwave were preferred to be selected in this mutation experiment.

#### 3.1.1. Mutation with Single Factors

**Figure 1:**
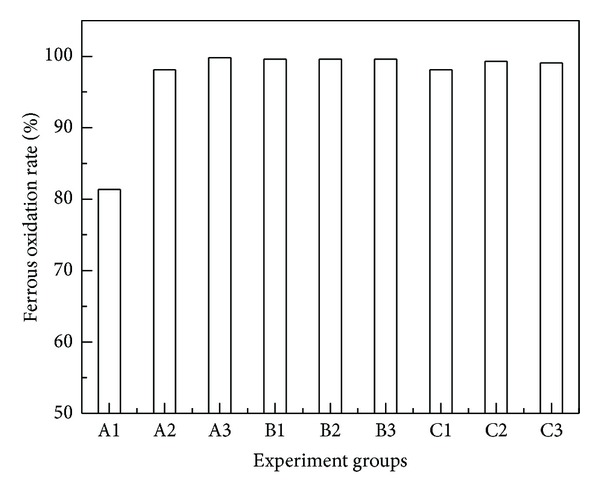
Mutation experiments with single factors. A1: UV 60 s, A2: UV 120 s, and A3: UV 180 s; B1: ultrasonic 10 min, B2: ultrasonic 20 min, and B3: ultrasonic 30 min; C1: microwave 10 s, C2: microwave 20 s, and C3: microwave 30 s.

Bacterial flora after mutation with single mutation factors were cultured in 250 mL flasks containing 100 mL 9 K liquid medium with ferrous iron. The initial density of cells was adjusted to about 1.0 × 10^7^ cells/mL. The results of ferrous iron oxidation rate were shown in Figure 1.

Most experimental groups had good effects. Especially, the mutation group A3 (UV 180 s) had the greatest effects on improving the rate of ferrous iron oxidation, which reached to 99.8% at 36 hours.

#### 3.1.2. Mutation with Combination of Mutation Factors

A problem that suffered in mutation process is repeated when using single mutagenic agents. It may cause bacteria to produce passive phenomenon and be hard to acquire the best mutation effect. Some researches revealed that several combined mutation techniques can increase mutation effect more efficiently than single mutation factors. El-Bestawy et al. [17] used ultraviolet irradiation combined ethidium bromide to obtain bacterial strains with capability of removing heavy metals at high efficiency, such as Cd (89.9–100%), Cr (87.3–99.7%) and Zn (47.7–100%). In this study, bacterial flora processed by combination of mutation factors were also conducted.

**Figure 2:**
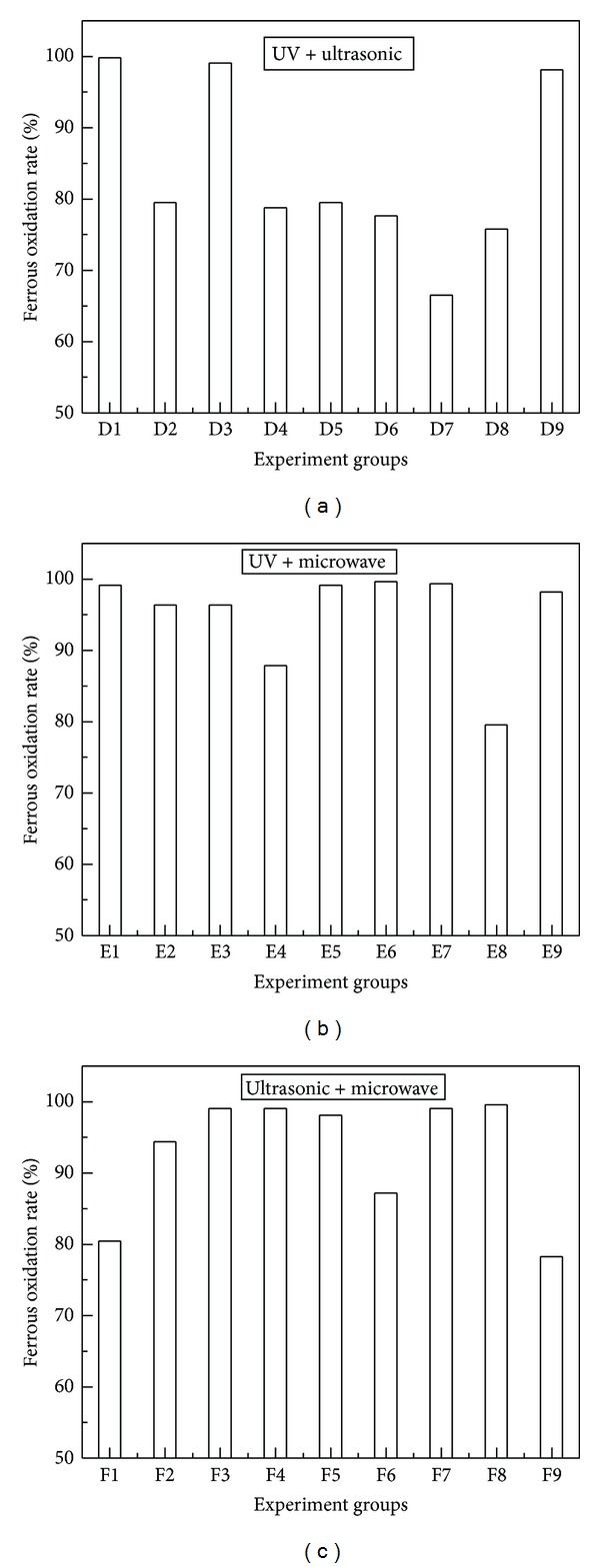
Mutation experiments with two combined mutation factors. (a) UV+ultrasonic (D1: UV 60 s + ultrasonic 10 min, D2: UV 60 s + ultrasonic 20 min, D3: UV 60 s + ultrasonic 30 min, D4: UV 120 s + ultrasonic 10 min, D5: UV 120 s + ultrasonic 20 min, D6: UV 120 s + ultrasonic 30 min, D7: UV 180 s + ultrasonic 10 min, D8: UV 180 s + ultrasonic 20 min, and D9: UV 180 s + ultrasonic 30 min), (b) UV + microwave (E1: UV 60 s + microwave 10 s, E2: UV 120 s + microwave 10 s, E3: UV 180 s + microwave 10 s, E4: UV 60 s + microwave 20 s, E5: UV 120 s + microwave 20 s, E6: UV 180 s + microwave 20 s, E7: UV 60 s + microwave 30 s, E8: UV 120 s + microwave 30 s, and E9: UV 180 s + microwave 30 s), (c) ultrasonic + microwave (F1: ultrasonic 10 min + microwave 10 s, F2: ultrasonic 20 min + microwave 10 s, F3: ultrasonic 30 min + microwave 10 s, F4: ultrasonic 10 min + microwave 20 s, F5: ultrasonic 20 min + microwave 20 s, F6: ultrasonic 30 min + microwave 20 s, F7: ultrasonic 10 min + microwave 30 s, F8: ultrasonic 20 min + microwave 30 s, and F9: ultrasonic 30 min + microwave 30 s).

Processed with two combined mutation factors. Bacterial flora after mutation with two combined mutation factors were cultured in 250 mL flasks containing 100 mL 9 K liquid medium with ferrous iron. The initial density of cells was adjusted to about 1.0 × 10^7^ cells/mL. The results of ferrous iron oxidation were shown in Figure 2.

**Figure 3:**
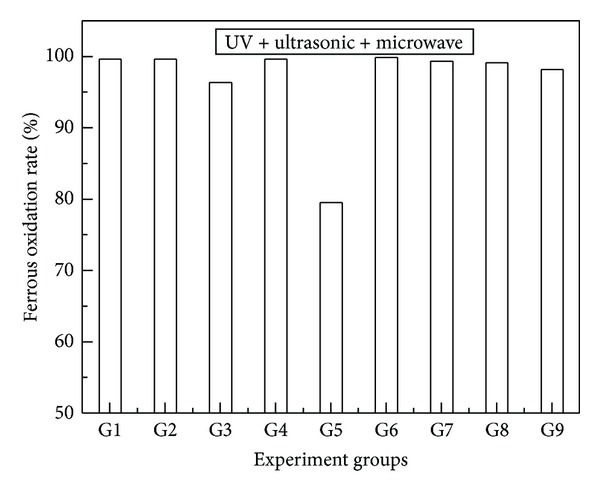
Mutation experiments with three combined mutation factors. (G1: UV 60 s + ultrasonic 10 min + microwave 10 s, G2: UV 60 s + ultrasonic 20 min + microwave 20 s, G3: UV 60 s + ultrasonic 30 min + microwave 30 s, G4: UV 120 s + ultrasonic 10 min + microwave 20 s, G5: UV 120 s + ultrasonic 20 min + microwave 30 s, G6: UV 120 s + ultrasonic 30 min + microwave 10 s, G7: UV 180 s + ultrasonic 10 min + microwave 30 s, G8: UV 180 s + ultrasonic 20 min + microwave 10 s, G9: UV 180 s + ultrasonic 30 min + microwave 20 s).

It was obvious that there were big fluctuations on mutation effects among different compound mutation groups.  Figure 2(a) showed ferrous iron oxidation results with combined mutation factors of UV and ultrasonic. Experimental groups from D1 to D9 indicated different mutation conditions. The best experimental group was D1 (UV 60 s + ultrasonic 10 min), which indicated that the bacterial flora had been processed by UV for 60 s, and then by ultrasonic for 10 min, the rate of ferrous iron oxidation could reach to 100% at 36 hours.  Figure 2(b) showed ferrous iron oxidation results with combined mutation factors of UV and microwave. The best experimental group was E6 (UV 180 s + microwave 20 s), the rate of ferrous iron oxidation could reach to 99.6% at 36 hours.  Figure 2(c) showed ferrous iron oxidation results with combined mutation factors of ultrasonic and microwave. The best experimental group was F8 (ultrasonic 20 min + microwave 30 s), the rate of ferrous iron oxidation could reach to 99.6% at 36 hours. Therefore, from Figure 3, it could be deduced that the experimental group D1 (UV 60 s + ultrasonic 10 min) had the greatest effect on improving the ferrous iron oxidation rate of bacterial flora.

Processed with three combined mutation factors. Bacterial flora after mutation with three combined mutation factors were cultured in 250 mL flasks, containing 100 mL 9 K liquid medium with ferrous iron. The initial density of cells was adjusted to about 1.0 × 10^7^ cells/mL. The results of ferrous iron oxidation were shown in Figure 3.

It showed that group G6 (UV 120 s + ultrasonic 30 min + microwave 10 s) had the best effect on improving the ferrous iron oxidation rate of bacterial flora, and it could reached to 99.8% at 36 hours.

### 3.2. Influences of Mutation on Bacterial Flora's Growth and Activity

Mutation technique had been wildly used to increase the production of industrially valuable compounds with microbial mutants [18]. And other researches had focused on adopting mutation technique to obtain microbial mutants owning more resistibility for heavy metals [19]. In this study, mutation was mainly adopted to improve the bacterial flora's growth and activity.

**Figure 4:**
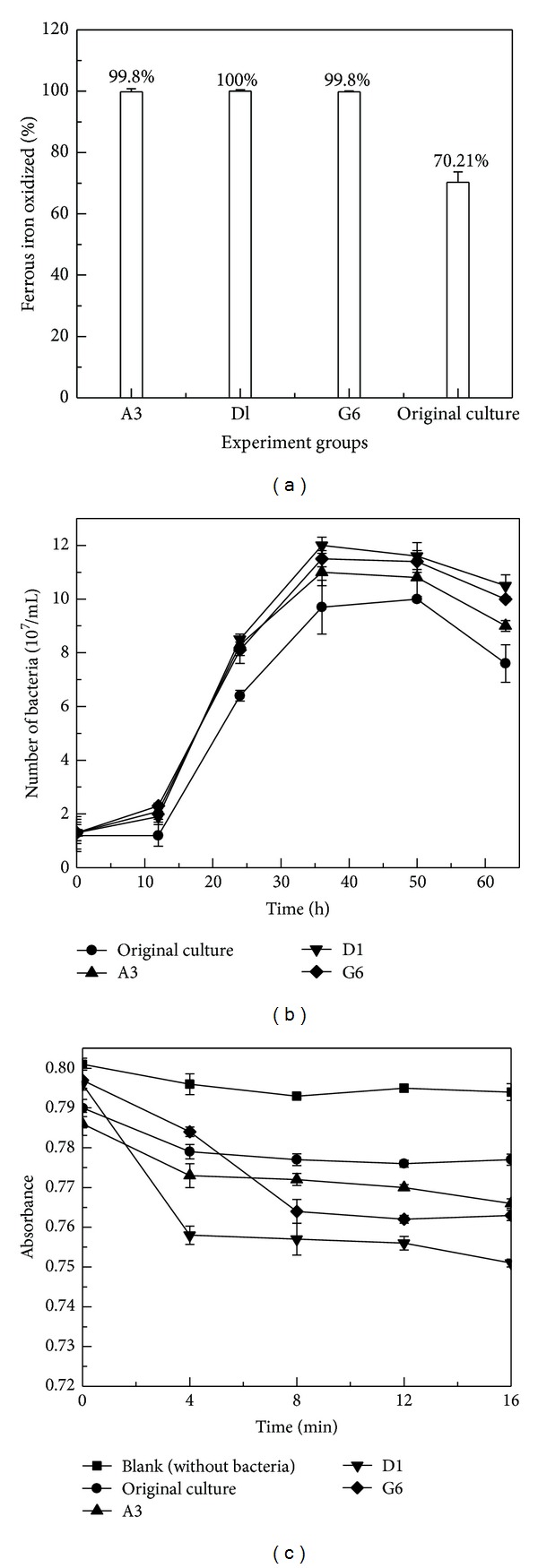
Various growth patterns of bacterial flora before and after mutation growing in 9 K medium with ferrous iron. (a) Ferrous oxidation rate, (b) growth curve, (c) total protein activity (A3: UV 180 s; D1: UV 60 s + Ultrasound 10 min; G6: UV 120 s + Microwave 10 s + Ultrasound 30 min).

Bacterial flora mutants, which were processed by three different mutation experimental groups such as A3 (UV 180 s), D1 (UV 60 s + ultrasonic 10 min), and G6 (UV 120 s + microwave 10 s + ultrasonic 30 min), respectively, were selected for further study. The untreated original culture and the mutants were cultivated in 250 mL flasks containing 100 mL 9 K liquid medium with ferrous iron, and the initial density of cells was adjusted to about 1.0 × 10^7^ cells/mL. The initial pH value of the culture was adjusted to 2.0 with 5 mol/L H_2_SO_4_. All flasks were performed at 40°C and 160 rpm in a rotary shaker. Various growth patterns of bacterial flora untreated and mutated growing in 9 K medium with ferrous iron were shown in Figure 4.

Figure 4(a) showed the results of ferrous iron oxidation of bacterial flora before and after mutation at 36 hours. For bacterial mutants, ferrous iron was almost oxidized completely within 36 hours. However, for the original culture, ferrous iron oxidation rate was only 70.21% at 36 hours. These results indicated that mutation could significantly improve the ferrous oxidation activity of this bacterial flora.

The growth curves of bacterial flora before and after mutation were shown in Figure 4(b). Results indicated that the growth of bacterial mutants was better than that of the original culture. And mutation treatments could obviously increase the density of cells in sequence of group D1 > G6 > A3. The density of cells reached to 1.2 × 10^8^, 1.15 × 10^8^, and 1.1 × 10^8^ cells/mL, increased by 20%, 15%, and 10% after mutation with group D1, G6, and A3, respectively. From this result, it could be known mutation could improve bacterial growth activity.

Furthermore, the activity of protein plays a key role in bacterial oxidation activity [20]. During autotrophic growth on iron, bacteria obtain energy through the oxidation of ferrous ion to ferric ion, and energy transduction is catalyzed by protein, such as rusticyanin, which participates in the ferrous oxidation pathway [21, 22]. In this study, the total protein activity was determined by a two-point inverse calibration method, and the results were shown in Figure 4(c). It could be seen that all the absorbance of the purple coordination compound continuously decreased as time increased. The absorbance values of bacterial mutants were always lower than that of the original culture and the blank (without bacteria) during the measuring time, which could indicate the total protein activity of bacterial flora mutants was improved. Meanwhile, the value of absorbance of different bacterial mutants was in sequence of group A3 > G6 > D1, which suggested that bacterial flora processed by mutation group D1 had the best total protein activity.

From these results, it could be deduced that the bacterial flora after mutation process with group D1 had the best ferrous oxidation activity, growth activity, and the total protein activity. It was selected as the experimental bacteria in further study of arsenic-rich gold concentrates bioleaching.

### 3.3. Bioleaching of Arsenic-Rich Gold Concentrates

**Figure 5:**
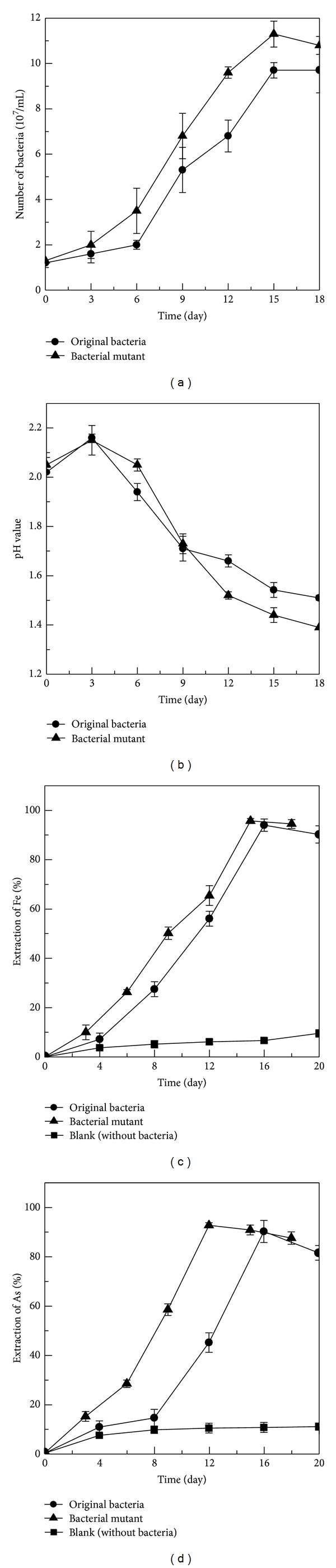
Various growth patterns of the original bacteria and bacterial mutant during arsenic-rich gold concentrates bioleaching. (a) Growth curve, (b) pH value, (c) extraction of Fe, and (d) extraction of As.

The original bacterial flora and the bacterial flora after mutation by method group D1 (UV 60 s + ultrasonic 10 min) were used for arsenic-rich gold concentrates bioleaching. The results in terms of changes in the density of cells, pH value, and extraction of elements Fe and As were shown in Figure 5.

As shown in Figure 5(a), bacterial mutants always grew better than the original bacteria during the whole bioleaching process, which was in accordance with the result of Dong et al. [23]. For the original bacteria, the density of cells was 9.7 × 10^7^cells/mL at 15 days. While for bacterial mutant, the density of cells reached to 1.13 × 10^8^ cells/mL at 15 days. However, the maximum concentration of cells was smaller than that growing in 9 K medium without arsenic-rich gold concentrates, because of high arsenic concentration inhibiting the bacterial growth.

Figure 5(b) showed pH values' variation during arsenic-rich gold concentrates bioleaching by the original bacteria and bacterial mutant. It indicated that pH value was fluctuating among the whole bioleaching process. At an earlier stage, pH value was uptrend. Then pH value continuously decreased as time increased both of the original bacteria and the bacterial mutant, down to 1.54 and 1.39 at 18 days, respectively. These results may attribute to that bacteria uses acid for its growth and reproduction at the beginning process (1) [24]. And then, the gold ore bioleaching involves in sulfur and sulfide minerals oxidation (2) to (4) [25, 26], which leads to a decrease in pH value [27]. These results were similar to the study of Zhou et al. [28]. Consider

(1) $$\begin{array}{c} 4{\text{Fe}}^{2+}+4{\text{H}}^{+}+{\text{O}}_{2}\overset{\text{bacteria}}{\to }4{\text{Fe}}^{3+}+2{\text{H}}_{2}\text{O} \end{array}$$

(2) 2FeAsS+2Fe3++4H2O+6O2  →2H3AsO4+4Fe2++2H++2SO42−

(3) $$\begin{array}{c} {\text{FeS}}_{2}+14{\text{Fe}}^{3+}+8{\text{H}}_{2}\text{O}\to {2{\text{SO}}_{4}}^{2-}+15{\text{Fe}}^{2+}+16{\text{H}}^{+} \end{array}$$

(4) $$\begin{array}{c} 2\text{S}+2{\text{H}}_{2}\text{O}+3{\text{O}}_{2}\overset{\text{bacteria}}{\to }4{\text{H}}^{+}+{2{\text{SO}}_{4}}^{2-} \end{array}$$

The extraction of total iron and arsenic leached by the original bacteria and bacterial mutant were shown in Figures 5(c) and 5(d). Firstly, all the figures indicated that arsenic-rich leaching by bacteria had better effect than that of leaching by acid. Then, bacterial mutant had better effect on the solubilization of total iron and total arsenic than that of the original bacteria. For leaching by the bacterial mutant, the extraction of total iron reached to 95.7% after 15 days. While, leaching by the original bacteria, the extraction of total iron was 85.8% after 15 days. A total of 9.9% extraction was improved by the bacterial mutant compared with original bacteria (Figure 5(c)). During the whole bioleaching process, the extraction of total arsenic by bacterial mutant was always higher than that by the original bacteria. For leaching by the bacterial mutant, the maximum leaching of arsenic reached to 92.6% after 12 days. When leaching by the original bacteria, the extraction of total arsenic was only 46.5% after 12 days. 46.1% of improvement was obtained by the bacterial mutant compared with that of the original bacteria (Figure 5(d)). At the later stage of bioleaching, the extraction of total iron and arsenic had a decrease trend, which might be due to the formation of secondary minerals such as jarosite and scorodite. Many researches indicated that in the process of mineral bioleaching the passivation layer was formed, adsorbed on the surface of mineral, and hindered the continuous bioleaching of mineral [25, 29–32].

### 3.4. XRD Analysis

**Figure 6:**
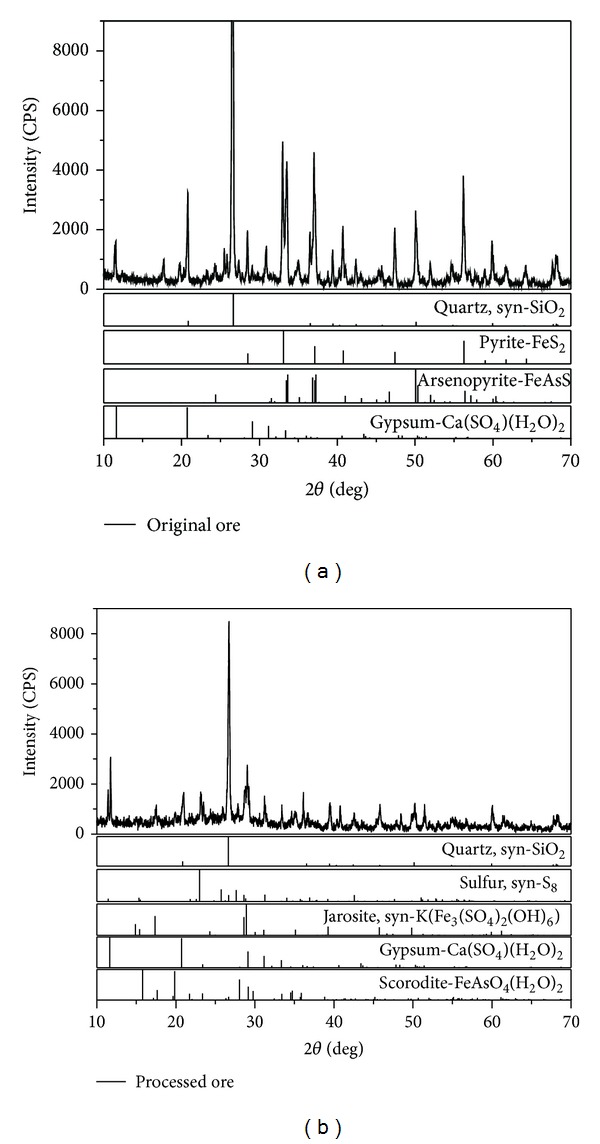
XRD patterns of the arsenic-rich gold concentrates' sample before and after bioleaching. (a) Original mineral sample, (b) leached residues.

In order to verify the formation of secondary minerals such as jarosite and scorodite on the surface of the arsenic-rich gold concentrates after bioleaching and oxidation, the X-ray diffraction (XRD) patterns of the original material and leached residues were further analyzed. The results were seen in Figure 6.

It showed that the main mineral phases were quartz (SiO_2_), sulfur (S_8_), jarosite (Fe_3_(SO_4_)_2_(OH)_6_), gypsum (CaSO_4_·2H_2_O), and scorodite (FeAsO_4_(H_2_O)_2_) in the residues (Figure 6(b)). Compared with the XRD patterns of the original concentrates (seen in Figure 6(a)), it could be deduced that arsenopyrite (FeAsS) and pyrite (FeS_2_) in the original mineral had been oxidized completely, and new materials such as sulfur (S_8_), jarosite (Fe_3_(SO_4_)_2_(OH)_6_), and scorodite (FeAsO_4_(H_2_O)_2_) had generated, which were substances that often form a passivation layer [25, 31, 32].

**Figure 7:**
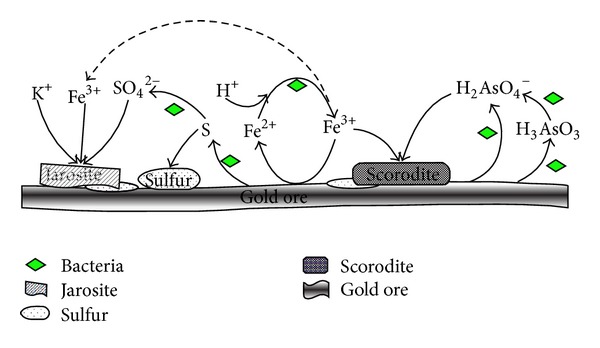
The formation of passivation layer on arsenic-rich gold concentrates surface.

On the base of XRD analysis results, the generation process of the passivation layer during bioleaching of the arsenic-rich gold concentrates was further derived, and a model was built in this study (seen in Figure 7).

From Figure 7, it could be seen that the gold concentrates were oxidized and generated H_2_AsO_4_^−^, Fe^3+^, S and SO_4_^2−^, which formed jarosite, scorodite, sulfur, and other substances covering in the mineral surface to form a passivation layer, wherein S layer was formed in the case of elemental sulfur which is excessive and not oxidized. The process might mainly refer to the following reactions [33–35]:

(5) 3Fe3++2HSO4−+K++6H2O  →KFe3(SO4)2(OH)6+8H+H3AsO4+Fe3++2H2O→FeAsO4·2H2O+3H+FeS2+2Fe3+→3Fe2++2SFeAsS+5Fe3++  3H2O→6Fe2++3H++H3AsO3+S

From the results it could be known that, for original bacteria and the mutant bacteria, the leaching process may be the same, and the leached residues may have the similar characteristics, but their leaching efficiency is different. Bacterial mutant could increase the extraction of iron and arsenic significantly and could shorten the leaching time.

## 4. Conclusion

In conclusion, the bacterial flora after mutation processed by combined mutation group D1 (UV 60 s + ultrasonic 10 min) had the best ferrous oxidation rate, the maximum density of cells, and the most total protein activity, and it had been selected for the further study of arsenic-rich gold concentrates bioleaching. Bioleaching results showed that the bacterial mutant could increase the extraction of iron and arsenic significantly and could shorten the leaching time. At the same time, XRD analysis results revealed the generation of new materials such as sulfur, jarosite, and scorodite covering in the gold concentrates' surface, which might form a passivation layer and hindered the continuous bioleaching of the gold concentrates. Further study focused on the community structure of the flora before and after mutation would be conducted. Succession of communities during the bioleaching process of the arsenic-rich gold concentrates will be the main study contents.

## Figures and Tables

**Table 1:** Elemental composition of arsenic-rich gold concentrates.

Element	Content (%)
Fe	19.3
As	9.3
S	20.4
Ni	0.024
Zn	0.266
Pb	0.08
Cu	0.086
Ca	2.9
Ag	0.006
Au	<0.001

## References

[1] Gao G, Li D, Zhou Y, Sun X, Sun W (2009). Kinetics of high-sulphur and high-arsenic refractory gold concentrate oxidation by dilute nitric acid under mild conditions. *Minerals Engineering*.

[2] Li Q, Li D, Qian F (2009). Pre-oxidation of high-sulfur and high-arsenic refractory gold concentrate by ozone and ferric ions in acidic media. *Hydrometallurgy*.

[3] Schmitz PA, Duyvesteyn S, Johnson WP, Enloe L, McMullen J (2001). Adsorption of aurocyanide complexes onto carbonaceous matter from preg-robbing Goldstrike ore. *Hydrometallurgy*.

[4] Zhu CL, Yang HY, Tang XG, Fan YJ, Tong LL (2010). Current status of studies on bacterial pre-oxidation and leaching of refractory gold ores with as. *Precious Metals*.

[5] Miller P, Brown A (2005). Bacterial oxidation of refractory gold concentrates. *Developments in Mineral Processing*.

[6] Cui R-C, Yang H-Y, Chen S, Zhang S, Li K-F (2010). Valence variation of arsenic in bioleaching process of arsenic-bearing gold ore. *Transactions of Nonferrous Metals Society of China*.

[7] Plumb JJ, Muddle R, Franzmann PD (2008). Effect of pH on rates of iron and sulfur oxidation by bioleaching organisms. *Minerals Engineering*.

[8] Liu Y-D, Wang X-B, Shi W-T, Yang Y, Zhang X (2010). Experimental research into the mutagenic mechanism of *Thiobacillus ferrooxidans*. *Transaction of Beijing Institute of Technology*.

[9] Xu A-L, Xia J-L, Zhang S, Yang Y, Nie Z-Y, Qiu G-Z (2010). Bioleaching of chalcopyrite by UV-induced mutagenized *Acidiphilium cryptum* and *Acidithiobacillus ferrooxidans*. *Transactions of Nonferrous Metals Society of China*.

[10] Kang J, Qiu G-Z, Gao J, Wang H-H, Wu X-L, Ding J-N (2009). Bioleaching of chalcocite by mixed microorganisms subjected to mutation. *Journal of Central South University of Technology*.

[11] Meng C, Shi X, Lin H, Chen J, Guo Y (2007). UV induced mutations in *Acidianus brierleyi* growing in a continuous stirred tank reactor generated a strain with improved bioleaching capabilities. *Enzyme and Microbial Technology*.

[12] Xia L, Zeng J, Ding J (2007). Comparison of three induced mutation methods for *Acidiothiobacillus caldus* in processing sphalerite. *Minerals Engineering*.

[13] Xia L-X, Tang L, Xia J-L (2012). Relationships among bioleaching performance, additional elemental sulfur, microbial population dynamics and its energy metabolism in bioleaching of chalcopyrite. *Transactions of Nonferrous Metals Society of China*.

[14] Yuan XW (2013). *Bioleaching of arsenic-rich gold ores with bacterial mixture before and after mutation and study of community shift [M.S. thesis]*.

[15] Xiao YS, Zhang XY (2001). Dotection of serum ferroxidase activity by two-point inverse calibration. *Jiangxi Journal of Medical Laboratory Sciences*.

[16] Johnson DB (1995). Selective solid media for isolating and enumerating acidophilic bacteria. *Journal of Microbiological Methods*.

[17] El-Bestawy E, Abou El-KHeir E, Abd El-Fatah HI, Hassouna SM (1998). Enhancement of bacterial efficiency for metal removal using mutation techniques. *World Journal of Microbiology and Biotechnology*.

[18] Mollania N, Khajeh K, Ranjbar B, Hosseinkhani S (2011). Enhancement of a bacterial laccase thermostability through directed mutagenesis of a surface loop. *Enzyme and Microbial Technology*.

[19] Zhao XW, Zhou MH, Li QB (2005). Simultaneous mercury bioaccumulation and cell propagation by genetically engineered *Escherichia coli*. *Process Biochemistry*.

[20] Zeng J, Geng M, Liu Y (2007). Expression, purification and molecular modelling of the Iro protein from *Acidithiobacillus ferrooxidans* Fe-1. *Protein Expression and Purification*.

[21] Ida C, Sasaki K, Ando A, Blake RC, Saiki H, Ohmura N (2003). Kinetic rate constant for electron transfer between ferrous ions and novel rusticyanin isoform in *Acidithiobacillus ferrooxidans*. *Journal of Bioscience and Bioengineering*.

[22] Yarzábal A, Duquesne K, Bonnefoy V (2003). Rusticyanin gene expression of *Acidithiobacillus ferrooxidans* ATCC 33020 in sulfur- and in ferrous iron media. *Hydrometallurgy*.

[23] Dong Y, Lin H, Wang H, Mo X, Fu K, Wen H (2011). Effects of ultraviolet irradiation on bacteria mutation and bioleaching of low-grade copper tailings. *Minerals Engineering*.

[24] Vilcáez J, Suto K, Inoue C (2008). Response of *thermophiles* to the simultaneous addition of sulfur and ferric ion to enhance the bioleaching of chalcopyrite. *Minerals Engineering*.

[25] Ahmadi A, Schaffie M, Petersen J, Schippers A, Ranjbar M (2011). Conventional and electrochemical bioleaching of chalcopyrite concentrates by moderately thermophilic bacteria at high pulp density. *Hydrometallurgy*.

[26] Ciftci H, Akcil A (2010). Effect of biooxidation conditions on cyanide consumption and gold recovery from a refractory gold concentrate. *Hydrometallurgy*.

[27] Plumb JJ, McSweeney NJ, Franzmann PD (2008). Growth and activity of pure and mixed bioleaching strains on low grade chalcopyrite ore. *Minerals Engineering*.

[28] Zhou H-B, Zeng W-M, Yang Z-F, Xie Y-J, Qiu G-Z (2009). Bioleaching of chalcopyrite concentrate by a moderately thermophilic culture in a stirred tank reactor. *Bioresource Technology*.

[29] Fantauzzi M, Licheri C, Atzei D (2011). Arsenopyrite and pyrite bioleaching: evidence from XPS, XRD and ICP techniques. *Analytical and Bioanalytical Chemistry*.

[30] Flemming RL, Salzsauler KA, Sherriff BL, Sidenko NV (2005). Identification of scorodite in fine-grained, high-sulfide, arsenopyrite mine-waste using micro x-ray diffraction (*μ*XRD). *Canadian Mineralogist*.

[31] He H, Xia J-L, Hong F-F, Tao X-X, Leng Y-W, Zhao Y-D (2012). Analysis of sulfur speciation on chalcopyrite surface bioleached with *Acidithiobacillus ferrooxidans*. *Minerals Engineering*.

[32] Chang-Li L, Jin-Lan X, Zhen-Yuan N, Yi Y, Chen-Yan M (2012). Effect of sodium chloride on sulfur speciation of chalcopyrite bioleached by the extreme thermophile *Acidianus manzaensis*. *Bioresource Technology*.

[33] Ahmadi A, Ranjbar M, Schaffie M (2012). Catalytic effect of pyrite on the leaching of chalcopyrite concentrates in chemical, biological and electrobiochemical systems. *Minerals Engineering*.

[34] Hol A, van der Weijden RD, van Weert G, Kondos P, Buisman CJN (2011). Processing of arsenopyritic gold concentrates by partial bio-oxidation followed by bioreduction. *Environmental Science and Technology*.

[35] Takatsugi K, Sasaki K, Hirajima T (2011). Mechanism of the enhancement of bioleaching of copper from enargite by thermophilic iron-oxidizing archaea with the concomitant precipitation of arsenic. *Hydrometallurgy*.

